# 中央型非小细胞肺癌立体定向放射治疗

**DOI:** 10.3779/j.issn.1009-3419.2018.05.10

**Published:** 2018-05-20

**Authors:** 于茗 万

**Affiliations:** 1 610041 成都，四川大学华西医院肺癌中心 Lung Cancer Center, West China Hospital, Sichuan University, Chengdu 610041, China; 2 610041 成都，四川大学华西医院肿瘤中心 Cancer Center, West China Hospital, Sichuan University, Chengdu 610041, China

**Keywords:** 立体定向放射治疗, 中央型, 肺肿瘤, Stereotactic body radiotherapy, Centrally, Lung neoplasms

## Abstract

已有的少数研究表明，早期不可手术非小细胞肺癌立体定向放射治疗肿瘤局控率可超过90%，患者中位生存时间和总生存率接近外科手术治疗。近年来立体定向放射治疗已成为Ⅰ期不可手术周围型非小细胞肺癌患者的推荐治疗。但中央型肺癌因其解剖位置多毗邻纵隔内重要脏器，常可伴有更明显的放疗副反应。中央型肺癌是否能进行立体定向放射治疗目前尚无统一定论，本文主要对中央型非小细胞肺癌立体定向放射治疗的定义、适应症、剂量分割方式、危及器官剂量-体积限制、放疗技术支持、副反应及立体定向质子治疗进行综述。

## Abstract (en)

A few study has proven that about 90% of local control rates might be benefit from stereotactic body radiotherapy (SBRT) for patients with medically inoperable stage Ⅰ non-small cell lung cancer (NSCLC), it is reported SBRT associated overall survival and tumor specific survival is comparable with those treated with surgery. SBRT has been accepted as the first line treatment for inoperable patients with peripheral located stage Ⅰ NSCLC. However, the role of SBRT in centrally located lesions is controversial for potential toxic effects from the adjacent anatomical structure. This paper will review the definition, indication, dose regimens, dose-volume constraints for organs at risk, radiation technology, treatment side effect of centrally located NSCLC treated with SBRT and stereotactic body proton therapy.

近年来，随着人口老龄化及肺癌筛查手段进步，早期肺癌检出率增高，临床上可见越来越多早期高龄非小细胞肺癌（non small cell lung cancer, NSCLC）患者，这部分患者由于器官功能退化手术耐受率低，约20% Ⅰ期NSCLC患者因一般状态差或慢阻肺、心脏病等合并症不能接受手术^[1]^，大于75岁的早期NSCLC患者手术接受率不到50%^[2]^。立体定向放射治疗（stereotactic body radiotherapy, SBRT）具有单次高等效生物剂量的特点，目前在早期NSCLC治疗中起着越来越重要的作用。已有的研究表明早期不可手术NSCLC立体定向放射治疗局控率可达80%-97%^[3]^，总生存率及肿瘤特异性生存率和手术相当^[4]^。美国国立综合癌症网络（National Comprehensive Cancer Network, NCCN）及欧洲临床肿瘤协会（European Society of Medical Oncology, ESMO）已将SBRT推荐于不能接受手术的早期周围型NSCLC患者^[5, 6]^。然而，中央型肺癌由于解剖位置毗邻纵隔内重要脏器结构，这部分患者接受SBRT后常伴有明显的放疗副反应^[7, 8]^。目前，中央型肺癌SBRT处于探索阶段，尚无统一结论。本文主要对中央型NSCLC立体定向放射治疗的定义、适应症、剂量分割方式、危及器官剂量-体积限制、放疗技术支持、副反应及立体定向质子治疗进行综述。

## 1 中央型肺癌

### 1.1 中央型肺癌基本定义NSCLC

SBRT有关研究对中央型肺癌的定义不一，主要有如下三种定义：①肿瘤侵及近端支气管树（包括隆突、左右主支气管及各叶支气管）各方向上2 cm以内区域，RTOG 0236研究采用了此种定义^[9]^。②肿瘤侵及近端支气管树各方向上2 cm以内区域，或计划靶区（planning target volume, PTV）包含纵隔胸膜或心包膜，如RTOG 0813研究选用的定义^[10, 11]^。③肿瘤侵及任何纵隔重要结构（支气管树、食管、心脏、臂丛神经、大血管、脊髓、膈神经、喉返神经）2 cm以内区域^[4, 12]^。根据中央型肺癌接受SBRT后出现的治疗相关副反应情况，为了尽量减少重要组织器官的放疗毒性，目前第三种定义在近年来的研究中应用最多。

### 1.2 超中央型肺癌

一些研究发现肿瘤和近端支气管树之间的距离与SBRT相关毒性反应发生率具有一定的关系^[13-15]^，因此，为了进一步探讨所有中央型肺癌患者接受SBRT后毒性有无差异，部分研究者又将大体肿瘤靶区（gross tumor volum, GTV）包含气管或近端支气管树^[16]^，或者PTV包含气管、近端支气管树、食管^[13, 17]^称为“超中央型（ultra-central）”。

Haseltine等^[15]^的研究中有4/18例肿瘤直接侵犯近端支气管树的患者在SBRT后7个月-9个月出现治疗相关死亡，其中3例接受9 Gy×5 F方案，1例接受10 Gy×5 F方案。肿瘤距离近端支气管树越近，SBRT后毒性反应及非肿瘤相关死亡风险越高^[13-15]^。Song等^[8]^发现6例肿瘤侵及主支气管和叶支气管的患者接受SBRT后均出现支气管狭窄，1例患者PTV包含主支气管，接受48 Gy/4 F分割方案SBRT后13个月出现出血及主支气管完全闭塞，最后死于治疗毒副反应；2例患者接受40 Gy/4 F分割方案后出现3级-4级肺毒性反应；另外3例患者接受60 Gy/3 F分割方案后仅有部分支气管狭窄但无明显呼吸系统症状。宾夕法尼亚大学Corradetti等^[18]^报道了一个“超中央型”肺癌患者接受SBRT案例，尽管其剂量分割方式、对正常组织限值与RTOG 0813一致，但该患者在接受50 Gy/5 F分割方案治疗后仍死于支气管坏死及出血。Rowe等^[19]^的研究中有1例SBRT相关死亡，患者肿瘤侵及左主支气管，左主支气管最大受量为54.2 Gy，最终于SBRT后10.5个月死于出血。

肿瘤直接侵及气管或近端支气管树是SBRT后出现治疗相关毒性的高危因素，具体原因不明，可能与近端支气管树肿瘤在放疗过程中改变了大气道及纵隔结构有关。有研究认为支气管树最大剂量点受量是出现支气管狭窄或支气管出血等副反应的重要剂量体积参数^[19]^。因此，这部分患者进行SBRT在分割方式、治疗毒性方面有别于其他的中央型肺癌患者，可能更适合选择减少单次放疗剂量，增加放疗次数的分割方式^[20]^。

## 2 中央型NSCLC立体定向放射治疗决策

中央型肺癌SBRT既往常报道有较高治疗相关副反应，尚缺乏高水平临床应用证据支持。近年来较多研究发现，选择合适的人群及剂量分割方案、对正常组织器官严格限量，同时结合先进的放疗技术使部分中央型肺癌患者可以考虑选择SBRT。

### 2.1 选择中央型肺癌SBRT治疗的合适人群

早期中央型非小细胞肺癌SBRT相关研究大多呈单中心，无统一纳入标准，多数参照RTOG 0813研究和Lungtech研究，主要依据肿瘤大小、分期、与周围重要危及器官毗邻关系来考虑是否进行SBRT。SBRT多选择中央区域仅含单个肿瘤的患者，RTOG 0813研究要求肿瘤长径 < 5 cm，仅纳入T1-2N0M0中央型NSCLC患者^[11]^。Lungtech研究还纳入部分T3患者（肿瘤大小≤7 cm，未位于纵隔内，且仅含一个病灶）^[21]^。Rowe等^[19]^发现有无限制性毒性与肿瘤大小明显相关（4.3 cm *vs* 2.9 cm, *P*=0.02）。Roach等^[22]^在2016年ESTRO年会上报道了一项多因素分析结果，结果发现是肿瘤大小而非肿瘤位置是局部失败的预后因素。肿瘤侵犯近端支气管树患者接受SBRT后常报道有严重治疗毒性，因此“超中心型”肺癌选择SBRT需要格外谨慎。Joesch等^[20]^认为肿瘤靠近隆突，中心气道浸润，肿瘤直径 > 4cm为早期肺癌选择SBRT的禁忌，对肿瘤≤4 cm，无中心气道浸润，距隆突 > 4 cm的患者大多（> 85%）推荐SBRT，既往接受过肺癌手术并非是选择SBRT的绝对禁忌症。

### 2.2 中央型肺癌SBRT剂量分割方案选择

中央型肺癌SBRT目前尚无标准剂量分割方案，最佳分割方式及最大处方剂量尚在探索中。SBRT疗效和生物等效剂量（biological effective dose, BED）之间具有一定的相关性，BED越低肿瘤局控率越差^[23]^，BED≥100 Gy是SBRT获得良好局控率的重要预后因素^[19]^。Onishi等报道了日本多个机构245例患者研究结果，发现BED10 > 100 Gy和 < 100 Gy亚组中局控率和3年OS均有显著性差异，分别为92% *vs* 74%（*P* < 0.05）、88% *vs* 69%（*P* < 0.05），但局控率在BED 10 > 120 Gy及 > 140 Gy组间无明显增加^[24]^。

一项Ⅱ期前瞻性研究^[25]^发现：中央型肺癌给予60 Gy-66 Gy/3 F分割方式治疗后，22例（27.3%）患者出现3级-5级放疗副反应。MD Anderson中心报道了100例中央型肺癌患者接受50 Gy/4 F或70 Gy/10 F剂量方案SBRT后结果，3年、5年总体生存率（overall survival, OS）分别为70.5%、49.5%，中位无进展生存期（progression-free survival, PFS）为42.5月，3年、5年无进展生存率分别为68.6%和63.6%，3年局控率和无远处转移率分别为96.5%、77.2%，未见4级或5级放疗副反应^[26]^。Kimura等^[27]^的Ⅰ期前瞻性结果显示60 Gy/8 F分割方案SBRT对Ia期中央型NSCLC是安全有效的。Davis等^[28]^分析了48例早期中央型肺癌患者接受平均48 Gy/4 F分割方案，中位BED10=105.6 Gy SBRT的研究结果：T1N0M0及T2N0M0患者2年OS分别为79%及32.1%（*P*=0.009），2年总体局控率为76.4%，未见3级及以上早期或晚期治疗副反应。

中央型肺癌因其解剖结构邻近重要危及器官，在考虑疗效的同时，平衡治疗毒性也很重要。因此，在尽量保证足够BED的情况下，可以选择减少单次剂量增加分割次数的治疗模式来减少晚期放疗毒性反应^[23]^。基于现有的研究，对能满足正常组织剂量体积限值的患者可以考虑接受45 Gy-50 Gy/4 F或者50 Gy-60 Gy/5 F分割方案，不能满足正常组织剂量体积限量的患者可以考虑60 Gy/8 F或70 Gy/10 F方案^[4]^。Daly等^[29]^分析了美国放疗医生对中央型肺癌SBRT分割方案的推荐情况：推荐50 Gy-55 Gy/5 F方案的占65%，推荐48 Gy-50 Gy/4 F方案占18%，推荐60 Gy/8-10 F方案的为9%，推荐54 Gy-60 Gy/3 F方案为7%。

肿瘤侵犯气管及近端支气管树的患者接受SBRT后易出现治疗相关毒性，这部分患者放疗建议采用更加保守的分割方式，荷兰阿姆斯特丹VU医疗中心对这部分患者常选择12×5 Gy和25×2.6 Gy分割方案^[30]^。在2017年ASTRO胸部肿瘤多学科会议中，Horne等^[31]^报道了40例孤立性肺门或纵隔淋巴结病灶接受SBRT后的随访结果，这部分患者接受中位分割方案为48 Gy/12 F治疗后，中位OS及PFS分别为22.7个月和13.1个月，2年局控率为87.7%，早期3级及以上毒性反应3例，晚期3级及以上毒性反应1例。

### 2.3 中央型肺癌SBRT对正常组织器官限量

SBRT对正常组织器官限量与传统放疗有所区别，目前并无统一的标准，具体限量情况应结合肿瘤的位置、分割方式及患者一般情况等综合考虑。MD Anderson中心和RTOG 0813研究的初步结果未见严重副反应，目前中央型肺癌SBRT对正常组织器官限量主要参照RTOG_0_813研究及MD Anderson中心经验^[4]^，主要对肺、气管、支气管树、大血管、食管、心脏、臂丛神经、脊髓等进行限量（表 1）。治疗毒性是限制中央型肺癌SBRT应用的主要因素，严格的剂量限制是中央型肺癌选择SBRT需要考虑的因素。

**1:** 中央型肺癌SBRT重要正常组织器官限量 Normal tissue dose-volume constraints for centrally located lung cancer with SBRT

	RTOG 0813			MD Anderson constrainsts					
	50-60 Gy/5 F			50 Gy/4 F			70 Gy/10 F		
Lung									
Total	V12.5 < 1, 500 cm^3^			MLD≤6 Gy			MLD≤9 Gy		
	V13.5 < 1, 000 cm^3^			V5≤30%			V40≤7%		
				V20≤12%					
				V30≤7%					
Ipsilateral	NA			MLD≤10 Gy			NA		
				V10≤35%					
				V20≤25%					
				V30≤15%					
Trachea	V18 < 4 cm^3^	Dmax < 105% PTV		V35≤1 cm^3^			V40≤1 cm^3^	Dmax < 60 Gy	
Bronchial tree	V18 < 4 cm^3^	Dmax < 105% PTV		V35≤1 cm^3^	Dmax≤38 Gy		V50 < 1 cm^3^	Dmax < 60 Gy	
Major vessel	V47 < 10 cm^3^	Dmax < 105% PTV		V40≤1 cm^3^	Dmax≤56 Gy		V50 < 1 cm^3^	Dmax < 75 Gy	
Esophagus	V27.5 < 5 cm^3^	Dmax < 105% PTV		V30≤1cm^3^	Dmax≤35 Gy		V40≤1 cm^3^	Dmax≤50 Gy	
Heart	V32 < 15 cm^3^	Dmax < 105% PTV		V20≤5 cm^3^	Dmax≤45 Gy		V45 < 1 cm^3^	Dmax≤60 Gy	
				V40≤1 cm^3^					
Brachial plexus	V30 < 3 cm^3^	Dmax < 32 Gy		V30≤0.2 cm^3^	Dmax≤35 Gy		V50 < 0.2 cm^3^	Dmax < 55 Gy	
Spinal cord	V22.5 < 0.25 cm^3^	Dmax < 30 Gy		V20≤1 cm^3^	Dmax≤25 Gy		V35≤1 cm^3^	Dmax < 40 Gy	
	V13.5 < 0.5cm^3^								
MLD: mean lung dose; Vx: volume of tissue exposed to x Gy or more; Dmax: maximum dose; NA: not available; PTV: planning target volume; SBRT: stereotactic body radiotherapy.							

### 2.4 中央型肺癌SBRT技术支持-呼吸运动管理及图像引导技术

SBRT实施主要基于呼吸运动管理及图像引导技术，通过4D-CT扫描定位得到不同呼吸时相的图像，继而产生内靶区（internal target volume, ITV），之后在ITV的基础上外扩一定范围生成PTV。若肿瘤位于呼吸动度较大的区域，定位时可以辅以呼吸门控或压迫式浅呼吸等方式减少呼吸动度。图像引导技术可以在治疗时对肿瘤位置进行再次确证，从而保证消融剂量落在肿瘤靶区内并在肿瘤边缘形成迅速的剂量跌落，现在常用CBCT在治疗过程中对靶区进行校正，近年来利用图像引导技术开展的自适应放疗进一步提高了放疗精确性^[23]^。

### 2.5 中央型肺癌SBRT的治疗副反应

中央型肺癌SBRT后的毒性反应是限制其应用的主要问题，近年来的研究多进行严格的正常组织剂量-体积限制及采用安全的分割方案，报道的治疗相关严重毒性反应有所减少。严重治疗相关毒性反应多发生在超中央型肺癌中，最常见的副反应有支气管狭窄、支气管出血、支气管坏死等。其他中央型肺癌SBRT后常见副反应为肺炎及胸壁疼痛，而肺功能减退、食管炎、食管狭窄、食管瘘，乏力等副作用相对少见^[26]^。SBRT前后使用抗VEGF制剂可能增加出血风险，应减少中央型肺癌SBRT和抗VEGF制剂联合应用^[15]^。

### 2.6 中央型肺癌SBRT近期重要前瞻性研究结果

近期重要的前瞻性研究主要有RTOG 0813及Lungtech研究。RTOG 0813主要探讨中央型NSCLC SBRT的有效性及最大耐受剂量，为剂量爬坡研究，分为5组，10 Gy/F、10.5 Gy/F、11 Gy/F、11.5 Gy/F、12 Gy/F，共5次，自2009年-2013年43个研究中心共纳入120例患者，Ⅰ期结果显示50Gy/5F组未见3级及以上副反应，60 Gy/5 F组出现7.2%的3级及以上毒性^[10]^。Ⅱ期结果显示60 Gy/5 F组（33例）及57.5 Gy/5 F组（38例）2年OS分别为72.7%及70.2%，与早期周围型肺癌结果相当。2年PFS分别为54.5%及52.2%，2年局控率分别为87.7%及89.4%。1年内早期毒性反应分别为4例及5例，1年后晚期毒性反应分别为5例及2例^[32]^。Lungtech研究目的是探讨早期中央型不可手术NSCLC SBRT有效性和毒性反应，采用60 Gy/8 F分割方式，主要研究终点为3年无疾病进展生存率，目前正在入组中，结果未出。

## 3 立体定向质子治疗（stereotactic body proton therapy, SBPT）

中央型肺癌紧邻重要危及器官，很难做到既取得满意疗效而又不出现明显的治疗副作用。随着调强质子治疗及图像引导质子治疗设备的出现，近年来逐渐开展了立体定向质子治疗。与光子治疗相比，质子治疗中央型肺癌能明显减少主动脉、支气管树、心脏、肺血管、脊髓等重要正常组织器官的平均最大剂量，质子治疗较光子有更好的剂量学优势，可以减少正常组织毒性反应^[33, 34]^。

Bush等^[35]^报道了111例早期NSCLC患者接受相对生物剂量（relative biological effectiveness, RBE）为51 Gy/10 F、60 Gy/10 F、70 Gy/10 F分割方案质子治疗后的结果，发现随着剂量增高，局控率和生存率均增高，且未见明显治疗相关毒性，因此对不能手术的早期中央型NSCLC患者选择70 Gy/10 F（RBE）分割方案的质子治疗是安全有效的；Register等^[36]^对比质子和光子SBRT计划后发现，质子治疗可以明显减少Ⅰ期中央型NSCLC及上肺癌正常组织受量。Chang等^[37]^报道了早期NSCLC（T1-3N0M0）质子治疗的Ⅰ期/Ⅱ期前瞻性研究结果，发现对肿瘤直径≥5 cm、上肺癌、中央型肺癌及肿瘤直接侵及危及器官患者，质子较光子SBRT更具有优势，35例患者接受RBE 87.5 Gy/35 F（BED=109.4 Gy α/β=10）分割方案质子治疗后1年、2年、3年、5年无进展生存率分别为80.0%、64.4%、53.6%、53.6%，5年无局部复发、无区域淋巴结复发及无远处转移率分别为85.0%、89.2%、56.0%，同时未见明显毒副反应。近来MD Anderson中心开展了一项对比SBRT和SBPT治疗早期中央型NSCLC的Ⅱ期临床试验（http://clinicaltrials.gov/show/NCT01511081），两组患者均接受50 Gy/4 F（RBE）分割方案，旨在对比两种治疗方式的治疗毒性及疗效，目前已入组完毕，但结果尚未公布。

质子治疗在中央型肺癌及上肺癌等肿瘤临近重要危及器官、靶区较大、多个肿瘤、既往照射野复发、肺功能较差的患者中较SBRT更具有优势^[35, 38, 39]^，但仍存在一些问题，如最佳剂量及分割方式不明确，现有报道多采取总RBE为51 Gy-87.5 Gy，单次RBE为1.8 Gy-16 Gy^[40]^；质子治疗计划复杂未得到普及应用；治疗费用昂贵，预计约为光子治疗费用的1.6倍-2.4倍^[38]^。

## 4 展望

虽然中央型肺癌SBRT常因治疗毒性反应受到应用限制，但通过严格正常组织剂量限制、采用合适的剂量分割模式及开展精准放疗技术可使SBRT应用更加广泛。质子治疗相较光子治疗可减少对正常组织的损伤，可作为中央型肺癌放射治疗的另一发展方向。部分研究报道了SBRT联合免疫治疗的满意疗效^[41-43]^，放射治疗在局部控制肿瘤的同时，也可通过远位效应激活机体抗肿瘤免疫反应发挥全身抗肿瘤作用^[44, 45]^，因此SBRT联合免疫治疗对部分晚期中央型肺癌患者也不失为可考虑的综合治疗模式。
